# British *Escherichia coli* O157 in Cattle Study (BECS): to determine the prevalence of *E. coli* O157 in herds with cattle destined for the food chain

**DOI:** 10.1017/S0950268817002151

**Published:** 2017-09-19

**Authors:** M. K. HENRY, S. C. TONGUE, J. EVANS, C. WEBSTER, I. J. McKENDRICK, M. MORGAN, A. WILLETT, A. REEVES, R. W. HUMPHRY, D. L. GALLY, G. J. GUNN, M. E. CHASE-TOPPING

**Affiliations:** 1 Epidemiology Research Unit (Inverness campus), Scotland's Rural College (SRUC), Kings Buildings, West Mains Road, Edinburgh EH9 3JG, UK; 2 Biomathematics and Statistics Scotland, James Hutton Institute, Invergowrie, Dundee DD2 5DA, UK; 3 RSK ADAS Ltd., Spring Lodge, 172 Chester Road, Helsby, Cheshire WA6 0AR, UK; 4 Immunity Division, The Roslin Institute, R(D)SVS, University of Edinburgh, Centre for Infectious Diseases, Easter Bush EH25 9RG, UK; 5 Centre for Immunity, Infection and Evolution, University of Edinburgh, King's Buildings, Edinburgh EH9 3JT, UK

**Keywords:** Bovine, epidemiology, *Escherichia coli* (*E. coli*) O157, estimating disease prevalence

## Abstract

*Escherichia coli* O157 are zoonotic bacteria for which cattle are an important reservoir. Prevalence estimates for *E. coli* O157 in British cattle for human consumption are over 10 years old. A new baseline is needed to inform current human health risk. The British *E. coli* O157 in Cattle Study (BECS) ran between September 2014 and November 2015 on 270 farms across Scotland and England & Wales. This is the first study to be conducted contemporaneously across Great Britain, thus enabling comparison between Scotland and England & Wales. Herd-level prevalence estimates for *E. coli* O157 did not differ significantly for Scotland (0·236, 95% CI 0·166–0·325) and England & Wales (0·213, 95% CI 0·156–0·283) (*P* = 0·65). The majority of isolates were verocytotoxin positive. A higher proportion of samples from Scotland were in the super-shedder category, though there was no difference between the surveys in the likelihood of a positive farm having at least one super-shedder sample. *E. coli* O157 continues to be common in British beef cattle, reaffirming public health policy that contact with cattle and their environments is a potential infection source.

## INTRODUCTION

Human infection with *Escherichia coli* (*E. coli*) O157 is a global concern, as infection can lead to kidney failure, neurological complications and haemolytic uraemic syndrome (HUS). HUS can be fatal, particularly in young, elderly or immunocompromised patients [1]. Worldwide, the incidence of HUS due to *E. coli* O157 infection has been reported at approximately 10% [2], with a 3–5% case-fatality rate [3], while the majority of those who survive suffer some degree of chronic renal function impairment [3]. Cattle and their environments are a reservoir of *E. coli* O157 [4–6]. Some strains produce verocytotoxin (verocytotoxigenic *E*. *coli* (VTEC) O157) and can be excreted in cattle faeces in high numbers, leading to the concept of super-shedding [7, 8]. Certain subtypes of *E. coli* O157, specifically those with the genetic marker encoding toxin *vtx 2*, are more likely to be associated with super-shedding in cattle and these also appear to pose the greatest risk for transmission to humans [8, 9]. There is also evidence that both verocytotoxin type and phage type are linked to, not only excretion levels in cattle but, disease severity in humans [10].

In 1998–2000 and 2002–2004, two national cross-sectional surveys in Scotland (SEERAD [11] and IPRAVE [12]) demonstrated the presence of *E. coli* O157 on approximately 20% of farms producing cattle for human consumption. A structured survey in England & Wales during 1999 estimated herd-level VTEC O157 prevalence to be 38·7% [13], while a 2003 convenience survey in England & Wales identified VTEC O157 on 32·2% of 255 farms [14]. Given the poor predictive value of a negative test result due to sporadic faecal shedding [15, 16], the advice from public health authorities has been to assume *E. coli* O157 are present in all cattle faeces [17]. Control of shedding from cattle has been suggested as a means to protect public health [9, 18], but is difficult to achieve.

Updated prevalence estimates are now required for Scotland and for England & Wales to contextualise the current risk to human health from cattle. As there is evidence that the primary VTEC O157 subtypes are changing in human infections in the UK [10], surveillance of cattle should continue, in order to confirm whether equivalent shifts have occurred in the cattle VTEC O157 population. If so, this would facilitate the development of measures to mitigate risk to humans.

The study was designed to conduct contemporaneous surveys on equivalent cattle populations in Scotland and England & Wales. Here we present the study methodology, descriptive analysis of the sampled farms, the herd-level and pat-level prevalence estimates obtained for *E. coli* O157 in British cattle destined for the food chain and the *vtx* frequencies found. This study provides the essential foundation for a number of further analyses and future investigative approaches.

## Methods

### Study design

The British *E. coli* O157 in Cattle Study (BECS) described in this manuscript is comprised of two surveys: one in Scotland and one in England & Wales.

In Scotland, the source population for the survey was the holdings that had participated in both of two earlier Scottish cross-sectional cattle surveys (SEERAD from 1998–2000 [11] and IPRAVE from 2002–2004 [12]) and still kept cattle aged between 1 and 2 years and/or cattle over 2 years without offspring – i.e. they were likely to still be producing cattle for slaughter. These were identified by matching the holding details from all the holdings sampled in the SEERAD [11] and IPRAVE [12] surveys to determine the subset of holdings that had been sampled in both. The postcode and farm names were then matched to official records of cattle numbers (June Agricultural Census 2012 and Cattle Tracing System (CTS) data from June 2013). The holdings sampled in the SEERAD and IPRAVE surveys were originally selected from a list comprising 3111 farms with cattle, randomly selected from 1997 Scottish Agricultural and Horticultural Census data [12].

The England & Wales survey was designed to be comparable to the Scottish survey. As there had been no previous survey, a slightly wider definition of eligible farms was adopted, to reduce the risk of excluding potentially eligible farms. In England & Wales, the source population comprised holdings containing either at least one (non-dairy breed) female aged 1 year or over, or at least one male (any breed) aged 1 year or over.

Sample sizes were estimated using reported prevalence from previous surveys (Scotland 20·5% [12] and England & Wales 39% [13]). Based on the proportion of herds positive and a sensitivity of 90%, sampling at least 110 farms in Scotland and 160 farms in England & Wales would provide 96% confidence that the true herd-level prevalence of *E. coli* O157 would fall within a tolerance range of 0·169 of the apparent prevalence estimated in these surveys. This would be similar to values estimated for SEERAD (0·179) and IPRAVE (0·161) [12].

The final sampling frame for Scotland contained 346 holdings. In England & Wales, the sampling frame was a random selection of 1280 holdings from a source population of 56 621. This number of holdings would ensure that, if a worst case scenario of a 1:8 participation response was assumed, we would be able to recruit the minimum number of holdings estimated in the sample size calculations above. Records were assigned a unique ID and the sampling frames were randomised before recruitment.

Recruiters and field samplers were trained according to a standardised protocol. There were two principal recruiters for each survey, with additional recruiters available if needed. Four samplers were available in Scotland and 10 in England & Wales.

Standard notification letters were sent to all farms 1 month before sampling started. Farms were then available for telephone recruitment if they had not opted out within 2 weeks.

To ensure objective recruitment, a recruitment software application was developed; this randomly selected one farm at a time from all eligible farms. From selection, it was the recruiter's responsibility to reach one of four potential outcomes: (1) contact made – further information requested; (2) farm recruited – passed to sampler for visit arrangement; (3) farm opted out; or (4) farm could not be reached – moved to a reserve list. The last outcome (4) followed three unsuccessful contact attempts. The reserve list would become available again had all farms been phoned without achieving the minimum sample size.

Recruited farms received a pack giving information on the study, details of the survey procedure, confidentiality, use of samples and data, information about *E. coli* O157 and a consent form. Farms were assigned a new unique ID once a sampling visit was arranged.

Sampling visits started in mid-September 2014 and were distributed as evenly as logistically feasible across geographical regions and over one calendar year. Each farm was visited once. The sample group was the group of non-breeding cattle closest to slaughter on the day of the visit. If mixed groups existed, the sampled group contained the cattle that met this definition. The sampling unit was a fresh faecal pat. Freshly voided discrete pats were preferentially sampled following the sampling protocol developed for the previous Scottish surveys [9, 17, 18]. The sample teams ensured that they did not sample from the same pat twice, nor from old, dried or desiccated pats. The number of pats taken from each group depended on group size and the sampling schedule from IPRAVE [12, 19, 20]. This gave 90% power to identify a sampled group as positive, if at least one animal were shedding *E. coli* O157.

For each sample, a 30 ml universal container was filled to just below the threaded portion with faeces taken from several locations on a fresh pat. Samplers preferentially targeted areas on the surface of the pat where mucus was apparent [21]. Samples were labelled and kept cool during transport to the laboratory.

At the sampling visit, a questionnaire was completed electronically through face-to-face interview. The questionnaire (available on request from the corresponding author) was adapted from the IPRAVE study. Questions covered aspects of farm demographics, management and health status. Most questions related to the farm although some were specific to the group of animals that was sampled. There was a different subset of questions for the sampled group, dependent on whether they were housed, or grazing, at the time of sampling.

### Approval

The Food Standards Agency approved and authorised informed consent documentation and the questionnaire. Personal data were handled in accordance with the Data Protection Act (1998).

### Case definition

A faecal pat was positive if *E. coli* O157 was detected using the laboratory methods below. A farm was positive if it contained at least one positive pat.

### Laboratory methods

*E. coli* O157 were isolated from 1 g of faeces per sample, using immunomagnetic separation methods previously described [22]. Enumeration of *E. coli* O157 was by limiting dilution method on CT-SMac agar plates and was performed in duplicate for each sample [23]. The limit of detection for enumeration was 100 colony-forming units per gram (CFU g^−1^). Polymerase chain reaction [24] was used to confirm the serogroup of the isolates as *E. coli* O157 and further characterise one *E. coli* O157 isolate per pat, according to the presence or absence of genes encoding toxins (*vtx*) 1 and 2. Isolates were sent to SERL (Scottish *E. coli* Reference Laboratory) for confirmation of identity, further subtyping of toxin genes and phage typing (results not included here).

### Statistical methods

Herd-level prevalence and pat-level prevalence were estimated using SAS software version 9.4 [25]. Other statistical analyses were performed using R version 3.2.3 [26] and additional R packages [27–31]. Surveys were analysed independently, except when stated otherwise. Univariate statistical comparisons of recruitment and questionnaire data within and between surveys were made using linear, generalised linear regression and analysis of variance models, likelihood ratio, Mann–Whitney, Fisher's exact and Pearson's *χ*^2^ tests and Pearson's product–moment correlation, as appropriate. The statistical significance level, *α*, was set at a value of 0·05 throughout.

#### Prevalence

Herd-level prevalence estimates for Scotland were calculated using generalised linear mixed models with a logit link function, fitted using marginal residual pseudo-likelihood (Proc Glimmix, SAS software [25]). This method was chosen as it provided a consistent framework for ongoing integrated modelling of the current data with the two historical Scottish prevalence surveys; this analysis will need to accommodate the use of different, but inter-related, ‘*G*-side’ covariance structures for different subsets of the data, reflecting the different sampling designs in different studies. This issue is of continued relevance because the sample for the current study was selected from the set of farms sampled in both previous studies, where one of these was not a simple random sample [12]. Thus, it is desirable to produce prevalence estimates for the most recent study which respect the sampling structures applied over the three successive surveys. ‘Farm’ and an effect to model the effect of spatial–temporal clustering in one of the previous studies were fitted as random effects. Mean estimates and confidence intervals (CI) were generated by back transforming from the model output on the logit scale. Scottish pat-level prevalence was modelled in a similar way.

Although there were no historical studies for England & Wales to be integrated into an analysis, and hence no requirement to model complex sampling structures, a similar implementation of the same approach to calculating farm and pat-level prevalence was adopted for these data. For England & Wales, a generalised linear mixed model was fitted, with a random ‘farm’ effect to model extra-binomial variability.

For all models, seasonal differences were estimated by incorporating ‘season’ into the model as a fixed effect, with statistical significance assessed using an *F* test in a type III test of fixed effects. Season was defined as: spring – March to May; summer – June to August; autumn – September to November; winter – December to February. Differences between surveys were assessed by applying a *t* test to an appropriate subset of the combined model outputs.

These calculations make no adjustment for the sensitivity and specificity of the assay therefore estimates can be considered as apparent prevalence throughout.

#### *E.coli* O157 count data and verocytotoxin genes

Descriptive statistics and count distributions were summarised for positive pats. Where samples were positive but counts could not be enumerated, these were classified as below enumeration limits (BEL). The probability of positive pats meeting the definition of super-shedder was calculated for two classifications – a count of either >10^3^ CFU g^−1^ faeces (SS3) or >10^4^ CFU g^−1^ faeces (SS4) [20] – and compared between surveys. At pat level, the presence of clustering due to a farm effect was assessed using a likelihood ratio test to compare models with and without a random ‘farm’ effect on outcomes of interest relating to the pat-level descriptive analysis (*vtx* production, SS3 and SS4 status). The odds of a farm having at least one pat that was SS3, SS4 or *vtx*-producing were compared between surveys.

#### Questionnaire data – descriptive analysis

Questionnaire data were summarised and described. Non-normally distributed continuous variables were transformed where appropriate. Categorical variables were treated as multi-level factors; remaining variables were dichotomous. Season was defined as stated earlier. Cattle management type had four levels: suckler beef (SB), specialist finisher (SF), dairy (D) and other (Oth).

The association between size category – defined as median total cattle greater or less than the median total cattle on sampled farms – and positive farm status was assessed using logistic regression.

#### Validity

The potential for bias with regard to farm herd size and spatial location was assessed. Registered herd size (obtained when identifying the sampling frames) was used for this comparison as data were available for all farms.

Median herd size of sampled farms was compared to the same measure for (i) the denominator population; (ii) all non-sampled farms; (iii) farms that opted out; (vi) farms that were not phoned and (v) farms that were reserved. Two definitions of denominator population were used in England & Wales – (a) farms available for phone recruitment and (b) all farms in the original sampling frame.

The potential for spatial bias was investigated using Nomenclature of Units for Territorial Statistics (NUTS) [32]. Based on the distribution of sampling frame farms across NUTS 2 regions, the proportion of sampled farms within each NUTS 2 region was compared with the expected proportion using Fisher's exact test. For England & Wales, many NUTS 2 regions contained very few farms; a simulated *P*-value was therefore reported for the England & Wales data. To check whether this might influence England & Wales results, Fisher's exact test with simulated *P*-value was also performed on the Scottish data, to compare with the calculated *P*-value.

## Results

### Farm visits

Sampling visits were completed by September 2015 in Scotland and by November 2015 in England & Wales. The England & Wales extension related to recruitment difficulties during spring 2015. One of the 111 Scottish farms visited was excluded from analyses due to ineligibility as was visited in error and had not been sampled in previous surveys. Three of the 163 England & Wales farms visited were excluded because transfer delays affected sample viability.

### Herd-level prevalence

**Fig. 1.**
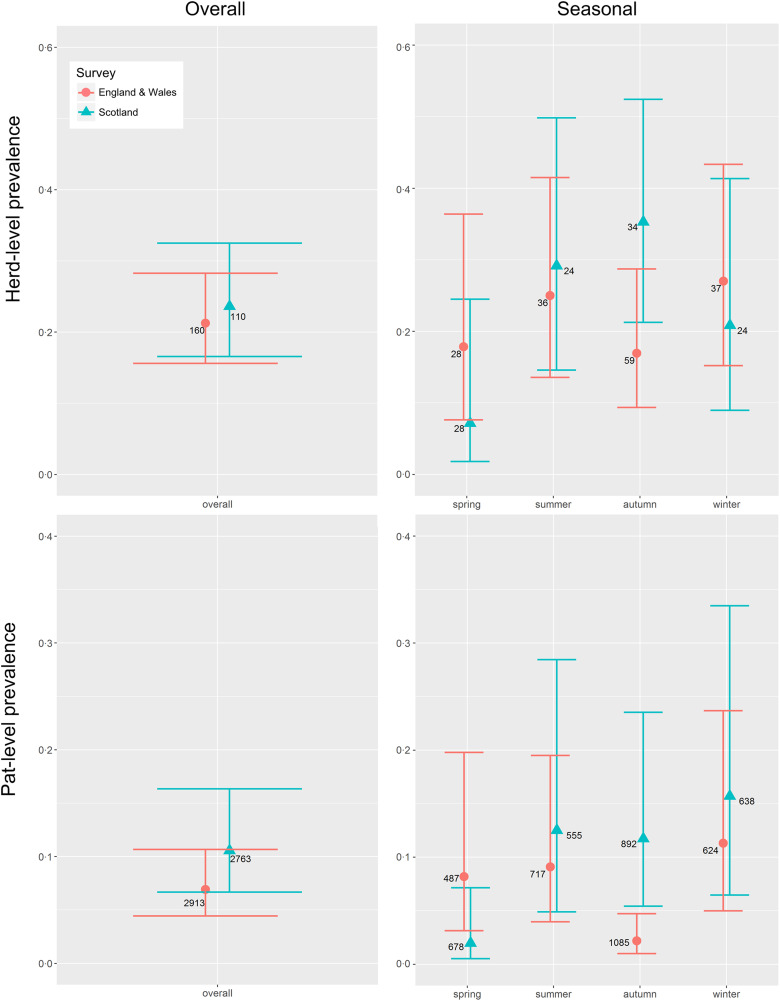
Mean seasonal prevalence estimates (solid triangles Scotland, solid dots England & Wales) including 95% CI (horizontal bars) for the herd-level and pat-level prevalence of *Escherichia coli* O157 in Scotland (blue) and in England & Wales (red) for farms sampled in Scotland (*n* = 110) and England & Wales (*n* = 160) between September 2014 and November 2015. Integer values beside each dot indicate the total number of farms or pats, as appropriate, sampled within each survey/season.

*E. coli* O157 was detected on 26 Scottish farms and 34 farms in England & Wales. The mean herd-level prevalence (95% CI) of *E. coli* O157 was estimated at 0·236 (0·166–0·325) and 0·213 (0·156–0·283), respectively (Table 1, Fig. 1). This difference was not statistically significant (*P* = 0·65).

**Table 1.** Estimates for mean herd-level and pat-level prevalence of Escherichia coli O157 for cattle farms sampled in Scotland and England & Wales between September 2014 and November 2015

Analysis level	*N*	Estimate	s.e.	Mean prevalence*	95% CI^†^
Herd					
Scotland	110	−1·174	0·225	0·236	0·166–0·325
England & Wales	160	−1·310	0·193	0·213	0·156–0·283
Pat					
Scotland	2763	−2·136	0·257	0·106	0·067–0·163
England & Wales	2913	−2·598	0·241	0·069	0·044–0·107

CI, confidence interval.

* 1/[1 + EXP(−estimate)].

† 1/[1 + EXP(−estimate ± (1·96 × s.e.))].

In Scotland, there was no difference in the number of herds sampled in each season (*P* = 0·36), whereas in England & Wales, the seasonal sampling distribution was not uniform (*P* = 0·001), with more samples taken in the autumn, the season with the lowest prevalence estimate (Fig. 1). Within surveys, there was no difference in seasonal herd-level prevalence in England & Wales (*P* = 0·92), but in Scotland spring estimates were significantly lower than autumn estimates (*P* = 0·02) (Fig. 1). Between surveys, autumn had the highest herd-level prevalence in Scotland but the lowest in England & Wales (*P* = 0·05) (Table 2, Fig. 1).

**Table 2.** Estimates for mean seasonal herd-level and pat-level prevalence of Escherichia coli O157 for cattle farms sampled in Scotland (N = 110) and England & Wales (N = 160) between September 2014 and November 2015

Analysis level		Mean prevalence – proportion (95% CI)		
	Season	Scotland	England & Wales	*P*-value
Herd	Spring	0·071 (0·017–0·250)	0·179 (0·076–0·364)	0·248
	Summer	0·292 (0·144–0·502)	0·250 (0·136–0·415)	0·724
	Autumn	0·353 (0·210–0·528)	0·169 (0·094–0·287)	0·053
	Winter	0·208 (0·088–0·418)	0·270 (0·152–0·433)	0·589
Pat	Spring	0·020 (0·005–0·072)	0·082 (0·031–0·198)	0·085
	Summer	0·125 (0·049–0·284)	0·091 (0·040–0·195)	0·604
	Autumn	0·117 (0·054–0·235)	0·022 (0·001–0·047)	0·003
	Winter	0·157 (0·065–0·335)	0·113 (0·050–0·237)	0·577

*P*-value shown is for *t* test of the difference between surveys

### Pat-level prevalence

The mean pat-level prevalence (95% CI) of *E. coli* O157 was estimated at 0·106 (0·067–0·163) for Scotland and 0·069 (0·044–0·107) for England & Wales (Table 1). The difference between Scotland and England & Wales was not statistically significant (*P* = 0·19). Within surveys, there was no difference in seasonal pat-level prevalence in England & Wales (*P* = 0·60), but in Scotland spring estimates were lower than estimates for the other seasons (*P* < 0·05) (Fig. 1). Between surveys, the pat-level prevalence in the autumn was low in England & Wales in comparison to Scotland (*P* = 0·003) (Table 2 and Fig. 1).

### *E. coli* O157 count data and verocytotoxin genes

Counts were determined for 287 *E. coli* O157-positive pats from Scotland and 234 from England & Wales. The distributions were highly skewed, with the median count in both surveys BEL. A subset of counts fell within SS3 and SS4 ranges (data not shown). At the farm level, there was no difference between surveys regarding the odds of a positive farm having at least one pat in either the SS3 or SS4 category (Supplementary Table S3). At the pat level, there was strong evidence of farm-level clustering within both surveys (*P* < 0·001). There was no difference between surveys in the probability of a positive pat having super-shedder status once farm-level clustering was accounted for (*P* = 0·97 for SS3 and *P* = 0·74 for SS4).

On 25 of 26 positive Scottish farms, at least one isolate of *E. coli* O157 produced *vtx*, compared with 29 of 34 positive farms in England & Wales (*P* = 0·22). At the farm level, there was no difference between surveys regarding the odds of a positive farm having at least one pat producing *vtx* (Supplementary Table S3). At the pat level, there was no difference found between surveys once farm-level clustering was accounted for (*P* = 0·84). In both surveys, the majority of positive isolates produced *vtx*2 alone; v*tx*1 appeared only with *vtx*2 (Supplementary Table S4).

### Descriptive statistics – questionnaire data

All Scottish farms completed questionnaires (*n* = 110). One questionnaire from England & Wales was incomplete (*n* = 159). Supplementary Tables S5–S8 give the univariable summary of questionnaire results for Scotland and England & Wales. No adjustment for multiple significance testing has been made.

The median ages of the youngest and oldest animals in the sampled groups, at 15 and 22 months in Scotland and 14 and 20 months in England & Wales, did not differ significantly (*P* = 0·18 and *P* = 0·28, respectively).

Scottish farms were larger (median total cattle at sampling) (*P* < 0·001), had more cattle aged 12–30 months (*P* = 0·015) and had larger sample groups (*P* < 0·001) than England & Wales farms. There were within-survey correlations between all three of these measures (Supplementary Table S8).

Few farms held organic status and distribution across management types was similar in both surveys (Supplementary Table S5). There was no difference between Scotland and England & Wales regarding health issues in the sampled group in the 2 weeks before sampling, or treatment being given in the 3 months before sampling (Supplementary Table S5). Scottish farms were more likely than those in England & Wales to have overwintered livestock owned by another keeper in the year before sampling (*P* = 0·002) and to employ farm workers (*P* < 0·001).

Fewer Scottish sampled groups were grazing at sampling than in England & Wales (*P* = 0·003). Compared to the autumn, sampled groups were more likely to be housed in spring in Scotland *(P* = 0·007), and during the winter in both surveys (*P* = 0·025 Scotland, *P* = 0·002 England & Wales). Bedding material was used in fewer Scottish housed groups than in England & Wales (*P* = 0·041) (Supplementary Table S6).

No differences were found in relation to questions asked specifically for grazing sample groups (Supplementary Table S7).

### Validity

Scottish sampled farms did not differ in median herd size from the denominator population: all farms in the original sampling frame (Supplementary Table S9).

In England & Wales sampled farms had larger median herd sizes than those in either definition of the denominator population: farms available for phone recruitment (a); or all farms in the original sampling frame (b) (*P* < 0·01) (Supplementary Table S9).

The 50% of the England & Wales farms that were largest in size, by total cattle numbers (i.e. above the median), were more likely to test positive for *E. coli* O157 than the 50% that were smallest in size (OR 3·652, *P* = 0·003). This effect was not seen in Scotland.

There was no difference in the proportional spatial distribution of Scottish denominator farms and sampled farms across NUTS 2 regions (*P* = 0·938). The same was seen for both definitions of denominator for England & Wales (*P* = 0·865 and *P* = 0·781). There was no difference in calculated *vs.* simulated *P*-value for this test on the Scottish data, therefore it was considered acceptable to report the simulated value for England & Wales.

## Discussion

In this study, for the first time, contemporaneous surveys have been completed in Scotland and England & Wales to obtain prevalence estimates for *E. coli* O157 in cattle destined for the food chain. The mean herd-level prevalence of *E. coli* O157 for the Scotland survey (0·236 (0·166–0·325)) did not differ statistically from that in the England & Wales survey (0·213 (0·156–0·283)). These estimates are similar to previous estimates for *E. coli* O157 in Scotland [12], but lower than previous estimates for England and Wales [13, 14].

The use of randomisation and the recruitment software removed much of the potential for recruitment selection bias, while the use of two main recruiters per survey with standardised protocols reduced the potential for recruitment bias due to inter-operator differences. There was no evidence for participation bias with regard to herd size, spatial location or sampling season in the Scotland survey. It can therefore be assumed that this is a valid estimate of current apparent prevalence for the source population. The original surveys were designed to be representative of the wider Scottish cattle population; whether this remains the case more than a decade later is open to question. Of the 447 Scottish farms that participated in both historical surveys 346 were still in business, with appropriate cattle officially recorded as present. The overall size and geographical distribution of the Scottish National Herd (SNH) has changed [33]. This could distort the current prevalence estimate if those changes are systematically associated with the likelihood of a farm being *E. coli* O157 positive – or factors that influence this – or with the reasons for the ineligibility of the no-longer-eligible subset [34]. The authors consider this to be unlikely, as there is no reason to believe that changes in the SNH are likely to have affected the survey population differently to the non-survey population, nor for them to be associated with *E. coli* O157-positive status. First, the main change in geographical distribution has been the contraction of the small proportion of the overall number of cattle in the SNH that are within dairy herds, both in numbers and geographically to the south west of Scotland. Second, the long-term gradual decline in overall cattle numbers has been evident since 1974 [33]. Thus, the mean herd-level prevalence for *E. coli* O157 is considered representative of the Scottish target population, i.e. those farms keeping cattle destined for the food chain.

There has been no previous comparable survey in England & Wales. As the categories and age groups for which data on cattle numbers are available have changed, the source population defined was the best achievable approximation to the eligibility requirements of the original Scottish survey [11]. Some farms included in the sampling frame may not have had cattle relevant to this study, making them ineligible. Unless they opted out, this would not have been discovered until they were contacted. Hence, the internal validity of the survey was assessed against two definitions of denominator farms.

As for the Scotland survey, the potential for recruitment bias in the England & Wales survey was minimised. There was no evidence for a spatial effect on participation, though smaller farms in England & Wales were both less likely to be randomly selected for phoning and also less likely to be sampled. Herd size distribution within this group did not differ statistically significantly from the group of farms from England & Wales that opted out initially. As it is unlikely that the lower likelihood of being sampled relates to the recruitment process, it is unfortunate that the reason for opting out when phoned and contacted was not recorded. This may have provided insight into whether this reflected ineligibility or disinterest. The mean herd-level prevalence of *E. coli* O157 may have been overestimated in England & Wales, given that, as a single variable, larger herds were more likely to test positive for *E. coli* O157 in this survey. Previously, herd size has been identified as a risk factor for Scottish farms being positive for *E. coli* O157, where – among positive groups – larger sample groups had lower mean within-group prevalence of shedding [11]. The opposite was seen in a survey of young cattle in England & Wales [14]. In both surveys presented here, there was a statistically significant difference in herd size between sampled farms and all farms that opted out. This highlights a potential recruitment challenge when conducting cross-sectional surveys that rely on single time-point records for cattle numbers and voluntary farmer participation, as it has implications for estimating prevalence of any condition that is known to be associated with herd size.

The statistically significant difference between the number of herds sampled across seasons in England & Wales is likely to be a direct result of recruitment issues encountered during the spring (Fig. 1). This meant that sampling extended into a second autumn period. If the autumn season were a known risk factor, or should sampling year influence the likelihood of a farm being positive, then this imbalance may have biased the overall England & Wales herd-level prevalence estimate. Previously, decreased herd-level prevalence in winter and a peak during the summer was found in Scottish herds, while housed status increased the mean shedding prevalence at group level [11, 35]. A longitudinal study of young cattle in England & Wales, however, found that winter was a risk period for shedding; it also corroborated the reduced risk for cattle at pasture [36]. In this study, winter had the highest herd-level and pat-level prevalence estimates for England & Wales, though seasonal differences were not statistically significant within our survey. Seasonal effects can be confounded by housing status due to management practices in the UK. In this study, the lower proportion of farms sampled during the spring in England & Wales may have decreased the herd-level prevalence estimate if housing were identified as a risk factor for positive farm status and groups were more likely to be housed during that season (therefore fewer housed groups were sampled than might have been expected), but this was not the case.

The prevalence estimate for England & Wales is substantially lower than those reported previously [13, 14]. Possible reasons for this include differences in how previous surveys defined an eligible farm, their sampling approach, the distribution of herds across management types and their seasonal distribution of sampling. The true prevalence may also have genuinely decreased. Having considered the potential differences between the current and previous approaches, the authors conclude that the estimated mean herd-level prevalence for *E. coli* O157 can be considered representative of the current England & Wales target population, i.e. those farms with cattle destined for the food chain.

This study demonstrates that *E. coli* O157 remains relatively widespread among British farms with cattle destined for the food chain.

No statistically significant difference was found between overall pat-level prevalence in Scotland and in England & Wales. The mean pat-level prevalence estimates from previous Scottish surveys were lower [12] than the current Scotland estimate, though this will be investigated further in another study. There are no previous pat-level estimates for a similar cattle population in England & Wales, although a sample-level prevalence of 7·7% for VTEC O157 has also been described in young cattle, based on rectal sampling [36]. Pat-level estimates will be a function of both the herd-level prevalence and the within-farm prevalence. This study was not designed to fully explore multi-level risk factors, although there is the potential for further analyses to investigate possible associations between demographic or management factors and within-farm prevalence of *E. coli* O157. Given that this study found strong evidence for farm-level clustering of super-shedder status and *vtx* status, this will be an important question to pursue.

Several factors may influence pat-level prevalence: temporal patterns of shedding by individual animals are known to vary [15, 16]; housing is associated with increased shedding [35]; there is known heterogeneity of distribution of *E. coli* O157 in pats [22] and it has not been possible to assess inter-operator differences within the current surveys, let alone between studies over time. In addition, climatic effects may affect the survival of the organism within pats [37, 38]. Any, some or a multi-factorial combination of these may have contributed to the overall pat-level prevalence estimates observed.

Despite the greater number of farms sampled in autumn in the England & Wales survey, the prevalence estimate for this season remains low compared with Scotland. Overall, the seasonal differences in herd-level and pat-level prevalence between the two surveys are interesting, particularly in the autumn and spring seasons. Further investigation of the *E. coli* O157 subtypes isolated from each survey may provide potential explanations for this observation.

A high proportion of positive farms from this study harboured isolates producing *vtx*, both in Scotland (0·962, 0·784–0·998) and in England & Wales (0·853, 0·682–0·945). However, six (one in Scotland; five in England & Wales) did not, which may reflect evolution of the persisting VTEC, as demonstrated in two Wisconsin dairy farms [39]. This finding, the lack of a statistical difference in *vtx* status between the surveys, plus the lack of a statistical difference in super-shedder status warrants more in depth investigation. The significance of super-shedder status (based on the SS3 definition) has recently been questioned [40]. There is also discussion about how to define a super-shedder (SS3 *vs.* SS4) [20, 21]. Regardless of whether it denotes a persistent characteristic of the individual animal or a phase through which all colonised cattle pass, super-shedding of *E. coli* O157 remains a public health issue through the introduction to the human environment of potentially harmful bacteria [17]. The classification performed for this study – into *vtx* 1 and 2 – will be augmented by investigating further subtyping of the toxin genes, the phage types and genetic structure of *E. coli* isolates collected via whole genome sequencing (WGS).

Over time, data from a 38 month long study in Swedish herds [41] demonstrated that, while previous positive VTEC O157:H7 status was a predictor for current status, for the majority of infected herds clearance of infection occurred within a limited period. Over a matter of years, data from the previous two Scottish surveys have demonstrated that prior *E. coli* O157 status at farm level is not a predictor of current status [42]. The design of BECS, where one of the objectives was to repeat sample a subset of Scottish farms for temporal analysis, provides a unique opportunity to further extend this investigation, which will be explored in future analyses.

These 2014/2015 cattle surveys have obtained isolates of *E. coli* O157 currently circulating in cattle in both Scotland and England & Wales, resulting in a unique collection. More detailed classification of collected strains and comparison with those from contemporaneous human clinical cases will give further insight into the relationship between circulating cattle and human isolates. With access to historic libraries of both cattle and human isolates for WGS, there is now the opportunity to investigate the evolution of this clonal type over the last two decades in the UK and elucidate the genetic determinants underlying zoonotic potential, such as variation in integrated prophages [43].

Only by determining the precise features of *E. coli* O157 that render it dangerous to humans and establishing the most reliable means of identifying cattle strains that pose the greatest risk will it be possible to target interventions appropriately within the cattle population and thus mitigate that risk to human health.

While providing the foundation for these further investigations, this work has demonstrated that *E. coli* O157 remains prevalent on British farms producing cattle for human consumption. Until further work to identify and characterise circulating strains is completed, public health messages should continue to outline the potential risk to human health from contact with cattle and their environment.

## Supplementary material

For supplementary material accompanying this paper visit https://doi.org/10.1017/S0950268817002151. click here to view supplementary material
