# Supplementary material for: British Escherichia coli O157 in Cattle Study (BECS): to determine the prevalence of E. coli O157 in herds with cattle destined for the food chain
Source: Epidemiol Infect. 2017 Sep 19;145(15):3168–79. doi: 10.1017/S0950268817002151 (PMC9148770; doi:10.1017/S0950268817002151)
Supplement: Supplementary file 1 [file S0950268817002151sup001.zip › Table_3-SI_revised.docx]

Table 3 - Supplementary Information: Odds ratio (comparison between surveys: for Scotland compared to England & Wales) of an E. coli O157 positive farm having at least one E. coli O157 supershedder pat (SS3* or SS4** definition) or at least one pat producing vtx.

| Analysis level | OR  [95% C.I.] | *P*-value |
| --- | --- | --- |
| At least one SS3* pat present | 1.0  [0.3 – 3.6] | 1 |
| At least one SS4** pat present | 2.0  [0.6 – 7.7] | 0.252 |
| At least one pat producing *vtx* | 5.2  [0.6 – 255.7] | 0.126 |

*an *E. coli* O157 count of greater than 10^3^ CFU g^-1^ faeces (SS3); ** an E. coli O157 count of greater than 10^4^ CFU g^-1^ faeces (SS4) as per [20]
