# Supplementary material for: British Escherichia coli O157 in Cattle Study (BECS): to determine the prevalence of E. coli O157 in herds with cattle destined for the food chain
Source: Epidemiol Infect. 2017 Sep 19;145(15):3168–79. doi: 10.1017/S0950268817002151 (PMC9148770; doi:10.1017/S0950268817002151)
Supplement: Supplementary file 1 [file S0950268817002151sup001.zip › Table_4-SI_revised.docx]

Table 4 – Supplementary Information: Description of positive E. coli O157 isolates according to vtx production, by survey.

| Analysis level | *vtx type* | Number (proportion) that had this *vtx* typ*e*  [95% CI] | |
| --- | --- | --- | --- |
| Survey |  | Scotland | England & Wales |
| *E. coli* O157 positive farms | | 26 | 34 |
| *E. coli* O157 positive farms with isolates | *vtx* negative | 1 (0.038)  [0.002 – 0.216] | 6 (0.176)  [0.074 – 0.352] |
|  | Any *vtx* present | 25 (0.962)  [0.784 – 0.998] | 29 (0.853)  [0.682 – 0.945] |
|  | *vtx1* only | 0 (0)  [0.000 – 0.160] | 0 (0)  [0.000 – 0.126] |
|  | *vtx2* only | 22 (0.846)  [0.643 – 0.950] | 23 (0.676)  [0.494 – 0.820] |
|  | *vtx1* and *vtx2* | 5 (0.192)  [0.073 – 0.400] | 7 (0.206)  [0.087 – 0.379] |
| *E.coli* O157 isolates | | 287 | 234 |
| *E. coli* O157 isolates | *vtx* negative | 1 (0.003)  [0.000 – 0.022] | 40 (0.171)  [0.126 – 0.227] |
|  | Any *vtx* present | 286 (0.997)  [0.978 – 1.000] | 194 (0.829)  [0.773 – 0.874] |
|  | *vtx1* only | 0 (0)  [0.000 – 0.016] | 0 (0)  [0.000 – 0.020] |
|  | *vtx2* only | 210 (0.732)  [0.676 – 0.781] | 133 (0.568)  [0.502 – 0.632] |
|  | *vtx1* and *vtx2* | 76 (0.265)  [0.215 – 0.321] | 61 (0.261)  [0.207 – 0.323] |

Note: proportion of farms does not sum to 1 per survey as farms could have several isolates producing different vtx types.
