# Supplementary material for: British Escherichia coli O157 in Cattle Study (BECS): to determine the prevalence of E. coli O157 in herds with cattle destined for the food chain
Source: Epidemiol Infect. 2017 Sep 19;145(15):3168–79. doi: 10.1017/S0950268817002151 (PMC9148770; doi:10.1017/S0950268817002151)
Supplement: Supplementary file 1 [file S0950268817002151sup001.zip › Table_5-SI_revised.docx]

Table 5 – Supplementary Information: Description by survey and comparison of questionnaire data for the variables common to **all sampled** groups.

|  |  | Number (proportion)  of farms | | *P*-value for difference  between surveys |
| --- | --- | --- | --- | --- |
| Survey |  | Scotland  N=110 | England & Wales  N=159 |  |
| Management type | Suckler Beef | 73 (0.663) | 109 (0.681) | 0.690 |
|  | Dairy | 14 (0.127) | 17 (0.106) | 0.699 |
|  | Specialist Finisher | 13 (0.118) | 18 (0.113) | 1 |
|  | Other | 10 (0.091) | 15 (0.094) | 1 |
| Sample season* | Spring | 28 (0.255) | 28 (0.175) | 0.128 |
|  | Summer | 24 (0.218) | 37 (0.225) | 1 |
|  | Autumn | 34 (0.309) | 59 (0.369) | 0.362 |
|  | Winter | 24 (0.218) | 37 (0.231) | 0.883 |
| Farm has organic status | | 5 (0.045) | 3 (0.019) | 0.278 |
| Cattle moved onto farm in the past 12 months | | 87 (0.791) | 121 (0.756) | 0.657 |
| Farm has shared a breeding bull in the past 12 months | | 19 (0.173) | 16 (0.100) | 0.098 |
| Livestock other than cattle purchased in the past 12 months | | 60 (0.545) | 88 (0.550) | 0.902 |
| Livestock overwintered in the past 12 months | | 33 (0.300) | 22 (0.138) | 0.002 |
| Livestock currently present on farm that are not owned by the farmer | | 19 (0.173) | 22 (0.138) | 0.492 |
| Organic waste from own farm spread in the past 12 months | | 85 (0.773) | 121 (0.756) | 0.884 |
| Organic waste from other farm(s) spread in the past 12 months | | 5 (0.045) | 9 (0.056) | 0.786 |
| Cows calve on the farm | | 97 (0.882) | 133 (0.831) | 0.379 |
| Cattle known to have access to water from a natural water source | | 75 (0.682) | 108 (0.675) | 1 |
| Employ farm workers in addition to main household | | 64 (0.582) | 51 (0.319) | <0.001 |
| Sample group had access to grazing | | 27 (0.245) | 68 (0.425) | 0.003 |
| Health problems seen in the sample group in the past 2 weeks | | 7 (0.064) | 7 (0.044) | 0.579 |
| Treatments used on cattle in the sample group in the past 3 months | | 41 (0.373) | 56 (0.350) | 0.796 |

*Values for sample season in England & Wales sum to 160 because this was known without completion of the questionnaire, as it relates to sampling date.
