# Supplementary material for: British Escherichia coli O157 in Cattle Study (BECS): to determine the prevalence of E. coli O157 in herds with cattle destined for the food chain
Source: Epidemiol Infect. 2017 Sep 19;145(15):3168–79. doi: 10.1017/S0950268817002151 (PMC9148770; doi:10.1017/S0950268817002151)
Supplement: Supplementary file 1 [file S0950268817002151sup001.zip › Table_6-SI_revised.docx]

Table 6 – Supplementary Information: Description by survey and comparison of questionnaire data for the variables that relate to the **housed** sample groups.

|  |  | Number (proportion)  of farms | | *P*-value for  difference |
| --- | --- | --- | --- | --- |
| Variable |  | Scotland  N=83 | England & Wales^  N=92 | between surveys |
| Type of housing  (more than one could be selected) | Straw courts | 63 (0.759) | 79 (0.859) | 0.121 |
|  | Slats | 11 (0.133) | 4 (0.043) | 0.056 |
|  | Byre | 7 (0.084) | 3 (0.033) | 0.195 |
|  | Other | 4 (0.048) | 7 (0.076) | 0.542 |
| Feeding changed in the past 2 weeks | | 21 (0.253) | 18 (0.196) | 0.371 |
| Location changed in the past 2 weeks | | 15 (0.181) | 16 (0.174) | 1 |
| New animals added in the past 2 weeks | | 10 (0.120) | 8 (0.087) | 0.619 |
| Bedding used where sample group housed* | | 73 (0.880) | 89 (0.967) | 0.041 |
|  | All old bedding removed prior to housing (N= *) | 63 (0.863) | 69 (0.775) | 0.163 |
|  | Wet bedding removed since housing (N= *) | 35 (0.479) | 53 (0.596) | 0.156 |
|  | New bedding added since housing (N= *) | 69 (0.945) | 84 (0.944) | 1 |
| Group had nose-to-nose contact with other cattle under 12 months | | 26 (0.313) | 20 (0.217) | 0.080 |

^ England & Wales data includes one group that had access to both housing and grazing
