# Supplementary material for: British Escherichia coli O157 in Cattle Study (BECS): to determine the prevalence of E. coli O157 in herds with cattle destined for the food chain
Source: Epidemiol Infect. 2017 Sep 19;145(15):3168–79. doi: 10.1017/S0950268817002151 (PMC9148770; doi:10.1017/S0950268817002151)
Supplement: Supplementary file 1 [file S0950268817002151sup001.zip › Table_7-SI_revised.docx]

Table 7 – Supplementary Information: Description by survey and comparison of questionnaire data for the variables that relate to the **grazing** sampled groups

|  |  | Number (proportion)  of farms | | *P*-value for  difference |
| --- | --- | --- | --- | --- |
| Variable |  | Scotland  N=27 | England & Wales^  N =68 | between surveys |
| Feeding changed in the past 2 weeks | | 6 (0.222) | 12 (0.176) | 0.578 |
| Location changed in the past 2 weeks | | 6 (0.222) | 15 (0.221) | 1 |
| Grazing shared with other cattle | | 5 (0.185) | 8 (0.118) | 0.509 |
| Grazing shared with other livestock species | | 4 (0.148) | 15 (0.221) | 0.573 |
| Grazing ground had been cut in the past 2 weeks | | 2 (0.074) | 3 (0.044) | 0.621 |

^England & Wales data includes one group that had access to both housing and grazing.
