# Supplementary material for: British Escherichia coli O157 in Cattle Study (BECS): to determine the prevalence of E. coli O157 in herds with cattle destined for the food chain
Source: Epidemiol Infect. 2017 Sep 19;145(15):3168–79. doi: 10.1017/S0950268817002151 (PMC9148770; doi:10.1017/S0950268817002151)
Supplement: Supplementary file 1 [file S0950268817002151sup001.zip › Table_8-SI_revised.docx]

Table 8 – Supplementary Information. Median values and correlation estimates for numbers of cattle in three groups: total cattle on farm, total cattle aged between 12 and 30 months, total cattle in the sample group. Pearson’s Product-Moment Correlation calculated on (log-transformed values +1) for each of the three independent variables.

|  | Scotland  N=110 | England & Wales  N=159 |
| --- | --- | --- |
| Median cattle (range) |  |  |
| on farm at sampling | 176  (6 – 849) | 85  (2 – 990) |
| 12-30 months | 31  (0 – 400) | 17  (0 – 260) |
| in sample group | 17  (1 – 90) | 14  (1 – 125) |
| Correlation [95% C.I.] (*P*) |  |  |
| between total cattle on farm and total cattle 12-30 months | 0.492  [0.338 – 0.622]  (<0.001) | 0.580  [0.467 – 0.675]  (<0.001) |
| between total cattle on farm and total cattle in sample group | 0.284  [0.103 – 0.448]  (0.003) | 0.535  [0.414 – 0.638]  (<0.001) |
| between total cattle 12-30 months and total cattle in sample group | 0.202  [0.015 – 0.375]  (0.034) | 0.485  [0.356 – 0.598]  (<0.001) |
