# Supplementary material for: British Escherichia coli O157 in Cattle Study (BECS): to determine the prevalence of E. coli O157 in herds with cattle destined for the food chain
Source: Epidemiol Infect. 2017 Sep 19;145(15):3168–79. doi: 10.1017/S0950268817002151 (PMC9148770; doi:10.1017/S0950268817002151)
Supplement: Supplementary file 1 [file S0950268817002151sup001.zip › Table_9-SI_revised.docx]

Table 9 – Supplementary Information. Validity: comparison of registered herd size for various groups in each survey.

| Analysis level | Group | n | Median  herd size | *P*-value  for within-survey difference in herd size compared to sampled farms |
| --- | --- | --- | --- | --- |
| Scotland | Sampled farms | 110 | 186 | - |
|  | (i) Denominator population | 346 | 155 | 0.198 |
|  | (ii) All non-sampled farms | 236 | 140 | 0.074 |
|  | (iii) Farms that opted out at either the preliminary letter, or phone stage | 99 | 103 | 0.002 |
|  | (iv) Farms that were not phoned | 100 | 162 | 0.775 |
|  | (v) Farms that were reserved | 37 | 166 | 0.839 |
| England & Wales | Sampled farms | 160 | 81 | - |
|  | (i) Denominator (a): Farms available for phone recruitment | 848 | 56 | 0.008 |
|  | (i) Denominator (b): All farms in the original sampling frame | 1264 | 55 | 0.004 |
|  | (ii) All non-sampled farms | 1101 | 51 | 0.001 |
|  | (iii) Farms that opted out at either the preliminary letter, or phone stage | 668 | 52 | 0.001 |
|  | (iv) Farms that were not phoned | 282 | 44 | <0.001 |
|  | (v) Farms that were reserved | 151 | 87 | 0.937 |
|  | Farms that were excluded from analyses due to sample transfer delay | 3 | - | - |
